# Integrating Internet multisource big data to predict the occurrence and development of COVID-19 cryptic transmission

**DOI:** 10.1038/s41746-022-00704-8

**Published:** 2022-10-28

**Authors:** Chengcheng Gao, Rui Zhang, Xicheng Chen, Tianhua Yao, Qiuyue Song, Wei Ye, PengPeng Li, Zhenyan Wang, Dong Yi, Yazhou Wu

**Affiliations:** Department of Health Statistics, College of Preventive Medicine, Army Medical University, Chongqing, 400038 China

**Keywords:** Viral infection, Live attenuated vaccines, Inactivated vaccines

## Abstract

With the recent prevalence of COVID-19, cryptic transmission is worthy of attention and research. Early perception of the occurrence and development risk of cryptic transmission is an important part of controlling the spread of COVID-19. Previous relevant studies have limited data sources, and no effective analysis has been carried out on the occurrence and development of cryptic transmission. Hence, we collect Internet multisource big data (including retrieval, migration, and media data) and propose comprehensive and relative application strategies to eliminate the impact of national and media data. We use statistical classification and regression to construct an early warning model for occurrence and development. Under the guidance of the improved coronavirus herd immunity optimizer (ICHIO), we construct a “sampling-feature-hyperparameter-weight” synchronous optimization strategy. In occurrence warning, we propose an undersampling synchronous evolutionary ensemble (USEE); in development warning, we propose a bootstrap-sampling synchronous evolutionary ensemble (BSEE). Regarding the internal training data (Heilongjiang Province), the ROC-AUC of USEE3 incorporating multisource data is 0.9553, the PR-AUC is 0.8327, and the *R*^2^ of BSEE2 fused by the “nonlinear + linear” method is 0.8698. Regarding the external validation data (Shaanxi Province), the ROC-AUC and PR-AUC values of USEE3 were 0.9680 and 0.9548, respectively, and the *R*^2^ of BSEE2 was 0.8255. Our method has good accuracy and generalization and can be flexibly used in the prediction of cryptic transmission in various regions. We propose strategy research that integrates multiple early warning tasks based on multisource Internet big data and combines multiple ensemble models. It is an extension of the research in the field of traditional infectious disease monitoring and has important practical significance and innovative theoretical value.

## Introduction

Early warning indicators of infectious diseases are an important measure in preventing and controlling infectious diseases. Accurate and timely perception of the transmission risk of infectious diseases is an important part of controlling their transmission and pandemic potential. In March 2020, the World Health Organization (WHO) confirmed the outbreak of corona virus disease 2019 (COVID-19) had reached pandemic status^1,2^. As of January 2022, the disease has caused more than 5 million deaths worldwide^3^. With the emergence of mutated strains such as Delta and Omicron, the cryptic transmission of COVID-19 cannot be ignored, and to do so may result in serious consequences^4,5^.

Cryptic transmission refers to infections that cannot be detected and reported in a timely manner by routine surveillance systems, including asymptomatic infections, mild respiratory symptoms, or sporadic pneumonia. Cryptic transmission has the characteristics of a short cycle and long incubation period, which retain the possibility of contagion and make conditions ripe for the spread of the disease; it is easy to miss the window of opportunity for pandemic prevention and control^6,7^. Because the clinical symptoms are not obvious, cryptic transmission often cannot be identified in time and becomes an obstacle to prevention and control measures^8^. In the early stages of the outbreak, limitations in initial diagnostic criteria and testing capacity left a large number of countries affected by cryptic transmission^9^. At present, the existing detection system often focuses on patients with obvious symptoms and does not pay enough attention to asymptomatic infections, resulting in a high risk of cryptic transmission^10^. If prevention and control measures and traffic control are not strictly enforced at this time and measures such as social distancing restrictions are not taken, cryptic transmission is likely to evolve into epidemic transmission. Once a pandemic begins, it will force a response by means of full staff nucleic acid detection, which greatly increases time and labour costs. In the stage of normalized prevention and control of the epidemic in China, there have been many cryptic transmission incidents in Heilongjiang and Shaanxi provinces, which have severely challenged prevention and control. However, the existing research on cryptic communication is limited to descriptive research^6–10^ and has not yet provided an effective early warning protocol of the occurrence and development of cryptic communication.

**Fig. 1:**
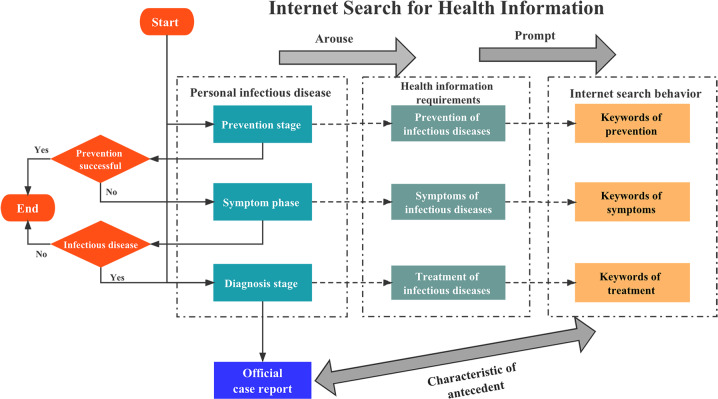
Leading features of Internet multisource big data. During the prevention, identification of symptoms, and diagnosis of infectious diseases, individuals will spontaneously generate health information search behaviour, thus generating Internet search behaviour data. Compared with official case monitoring, Internet search data are proactive and relevant.

Traditional infectious disease monitoring only collects data at the diagnosis stage, which leads to problems such as incomplete coverage and weak timeliness. There are limited early warning and monitoring capabilities for newly discovered unexplained infectious diseases. Currently, with the rapid development of health service informatization and Internet big data technology, the way people obtain health information and mine data has gradually changed^11^. The Internet is the source of much health-related data, which have high application value. Better integration and utilization of Internet big data can improve the sensitivity and timeliness of infectious disease early warning systems, which is an optimization and extension of traditional infectious disease early warning monitoring research^12^. At present, there are some studies on tracking infectious diseases using Internet search data^13–16^, and the results have shown that there is a correlation between COVID-19 and specific Internet search words, as shown in Fig. 1.

In the selection of Internet big data, previous studies mostly focused on Internet retrieval data, such as the Google Flu Trends (GFT)^17–20^. Nevertheless, it cannot be ignored that individuals’ Internet search behaviour is not entirely spontaneous and may be prompted by external factors (such as media information). Simply using Internet retrieval data cannot fully reveal the information search behaviour of groups on the Internet and may increase the risk of overfitting during the prediction process^21–23^. In previous studies, some scholars have used the GFT to analyze the incidence rate of flu and have detected an abnormal phenomenon in which the search index has increased but no local epidemic has occurred^24–26^. The abnormality was due to the interference effect caused by online media data. Hence, we put forward a solution of Internet multisource big data; that is, we propose combining the comprehensive application of Internet retrieval data, population migration data, and Internet media data to provide an early warning mechanism for the occurrence and development of COVID-19 cryptic transmission.

To ensure the accuracy and generalization of the early warning effect, both comprehensive data preparation and an appropriate early warning model need to be constructed. If expressed in statistical language, the occurrence of cryptic transmission can be regarded as a classification, with a situation of occurrence or nonoccurrence, while the development can be regarded as a regression to fit the epidemic trend. Machine learning methods are often used in the early warning systems for infectious diseases, and the key to their application lies in the selection of features and parameters^27,28^. A traditional method needs to select features first and then perform parameter optimization; the operation process is cumbersome, and the optimization efficiency is low. In addition, 0–1 programming was often selected in previous methods, and the standard of feature selection was too simple to adjust for practical problems, which easily falls into the problem of local optimization^29–31^. Hence, we regard the selections of features and parameters as optimization problems and improve the accuracy and synchronization on the basis of previous research. For feature selection, we innovatively propose the concepts of feature probability and feature scale to accurately obtain the optimal feature subset. In terms of parameter selection, we use the synchronous optimization method to improve the efficiency of parameter estimation. In addition, in ensemble learning, data balance processing and basic learner weights are also key to the application^32–34^. In traditional research, the steps to determine the data sampling method and the weight of the base learner are cumbersome and depend on subjective experience, which is difficult to determine accurately and quickly.

Hence, we regard the steps of data sampling, feature selection, hyperparameter optimization, and basic learner weight determination as optimization problems and improve accuracy and synchronization on the basis of previous research. Synchronous optimization is beneficial to efficiently discover key features, select appropriate hyperparameters and weights, and perform sampling processing according to data characteristics, thereby improving computational efficiency and model accuracy. Regarding data sampling, we adaptively select the appropriate dataset for each base learner. In feature selection, we propose the concepts of feature probability and feature scale to accurately obtain the optimal feature subset. In hyperparameter optimization, we use the synchronous optimization method to improve the efficiency of parameter estimation. In terms of weight determination, we adaptively select the weights of each base learner and then combine them into an optimal ensemble learning method. In addition, we assume that the influence of online media data can induce residuals and then perform an ensemble study using a combination of linear and nonlinear models^35^.

In addition, in the synchronous optimization process, to improve accuracy and reduce processing time, we chose a metaheuristic algorithm as the optimization strategy. However, traditional algorithms often have problems such as slow convergence speed, and they easily fall into local optimum^36,37^. Research on optimization algorithms with accurate solution methods, and robust computing power is still an important research direction for feature and parameter selection^38,39^. Hence, we also propose an improved coronavirus herd immunity optimizer (ICHIO), which aims to perform optimization efficiently and accurately.

In this study, we collect Internet multisource big data (including retrieval, migration, and media data) and propose comprehensive and relative application strategies to eliminate the impact of national and media data. The proposed method not only studies the occurrence of cryptic transmission but also predicts the development trend of cryptic transmission. Under the guidance of the improved coronavirus herd immunity optimizer (ICHIO), we construct a “sampling-feature-hyperparameter-weight” synchronous optimization strategy. In occurrence warning, we propose an undersampling synchronous evolutionary ensemble (USEE); in development warning, we propose a bootstrap-sampling synchronous evolutionary ensemble (BSEE). Regarding the external validation data (Shaanxi Province), the ROC-AUC and PR-AUC values of USEE3 were 0.9680 and 0.9548, respectively, and the *R*^2^ of BSEE2 was 0.8255. We propose strategy research that integrates multiple early warning tasks based on multisource Internet big data and combines multiple ensemble models. It is an extension of the research in the field of traditional infectious disease monitoring and has important practical significance and innovative theoretical value.

## Results

We applied the processed data to two tasks, classification and regression. The classification task determines whether cryptic propagation occurs, and the regression task fits the development trend of cryptic propagation. We proposed an USEE in classification and a BSEE in regression. In the effect evaluation, we performed ablation studies with various data and methods.

### Warnings of the occurrence of cryptic transmissions

In comparing various data, when the combined data were included, the classification performance was significantly better than those of the other two models. After constructing USEE, we divided it into USEE1, USEE2, and USEE3 based on the various data inclusion methods. The distinction between these models is in the inclusion of various types of network big data, as shown in Table 1. Table 2 shows a comparison of the prediction effects of the three models.

**Table 1:** Data selection of the classification models.

Data	Features	USEE1	USEE2	USEE3
Baidu search index	38	Regional/National	Regional/National	Regional/National
Baidu migration index	2	Regional	Regional	Regional
Baidu information index	8	–	Regional/National	Regional/National
TikTok composite index	8	–	–	National
Stock sector index	14	–	–	National

“Regional” represents regional Internet data, “national” represents national Internet data, and “regional/national” represents the ratio of regional and national Internet data. *USEE* under-sampling synchronous evolutionary ensemble. Affected by data sources and characteristics, the Baidu migration index can only choose regional data, and the TikTok composite index and stock index can only choose national data.

**Table 2:** Comparison of the classification effects of various data combinations.

Model	Dataset	PRE	SEN	SPE	ACC	F1	ROC-AUC	PR-AUC
USEE1	Training	0.6588	0.9825	0.8535	0.8824	0.7887	0.9791	0.9112
	Testing	0.6364	0.7368	0.8788	0.8471	0.6829	0.8086	0.6103
USEE2	Training	0.7368	0.9825	0.8990	0.9177	0.8421	0.9890	0.9303
	Testing	0.6667	0.8421	0.8788	0.8706	0.7442	0.8445	0.7846
USEE3	Training	0.8594	0.9649	0.9546	0.9569	0.9091	0.9908	0.9480
	Testing	0.7619	0.8421	0.9242	0.9059	0.8000	0.9553	0.8327

*USEE* under-sampling synchronous evolutionary ensemble. *PRE* precision, *SEN* sensitivity, *SPE* specificity, *ACC* accuracy, *F1* F1-Score, *ROC-AUC* receiver operating characteristic-area under curve, *PR-AUC* precision recall-area under curve.

**Fig. 2:**
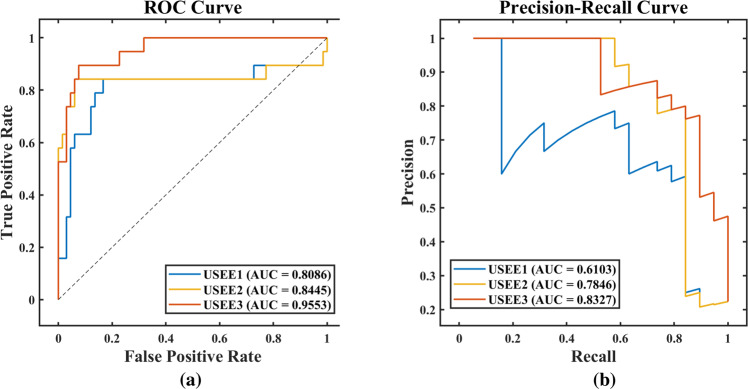
Comparison of the classification effects of various data combinations. Only test set results are shown. **a** ROC Curve, **b** Precision-Recall Curve.

**Fig. 3:**
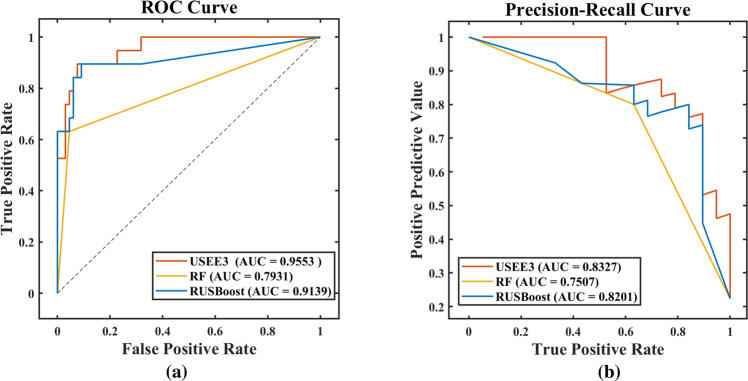
Comparison of the classification effects of various methods. Only test set results are shown. **a** ROC Curve, **b** Precision-Recall Curve.

In the comparison of various methods, the classification performance of our USEE was significantly better than that of traditional ensemble methods, as shown in Table 3. The ROC curve and PR curve of the classification effect evaluation also confirmed this conclusion, as shown in Figs. 2 and 3. Hence, USEE3 has the best prediction accuracy on the test set.

**Table 3:** Comparison of the classification effects of various methods.

Model	Dataset	PRE	SEN	SPE	ACC	F1	ROC-AUC	PR-AUC
USEE3	Training	0.8594	0.9649	0.9546	0.9569	0.9091	0.9908	0.9480
	Testing	0.7619	0.8421	0.9242	0.9059	0.8000	0.9553	0.8327
RF	Training	1.0000	0.9649	1.0000	0.9922	0.9821	0.9825	0.9864
	Testing	0.8000	0.6316	0.9546	0.8824	0.7059	0.7931	0.7507
AdaBoost	Training	–	0	1	0.7765	–	0.3912	–
	Testing	–	0	1	0.7765	–	0.3788	–
RUSBoost	Training	1.0000	1.0000	1.0000	1.0000	1.0000	1.0000	1.0000
	Testing	0.8000	0.6316	0.9546	0.8824	0.7059	0.9139	0.8201

We analyzed the reasons for the differences in the effects of each method. The traditional method does not introduce a data balance strategy, which is not suitable for processing our imbalanced dataset, and the prediction results are relatively inaccurate, especially the output of AdaBoost, which is negative. As a result, some of the evaluation indicators are abnormal. The USEE, which introduced the undersampling strategy, has better fitness in the face of imbalanced data and can solve the problem of data imbalance without changing the original data.

**Fig. 4:**
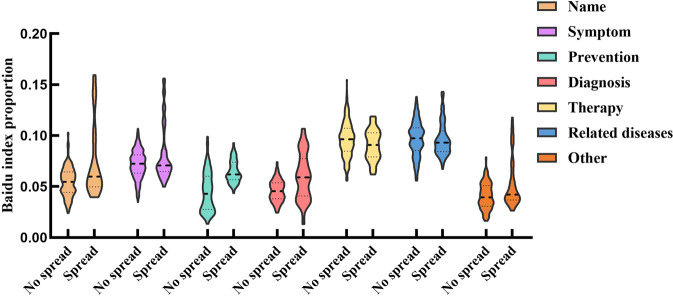
Distribution comparison of various types of keyword search indexes. The distribution characteristics of various types of keyword search indexes in the transmission and nontransmission stages. The results show that the search index in the transmission stage has more outliers, and the search index in the transmission stage is significantly higher than that in the nontransmission stage, especially in the three categories of name, prevention, and diagnosis.

A violin chart of each type of keyword search index was drawn. The results show that the search index has more outliers in the epidemic transmission stage, and the index level is generally higher, as shown in Fig. 4.

### Warnings of the development of cryptic transmissions

To study the early warning of the development of cryptic transmission, the period of cryptic transmission in Heilongjiang Province (76 days in total) was selected for research, with 80% (61 days) of data as the training set and the remaining 20% (15 days) as the test set. The preorder of the base learner incorporates network retrieval data and population migration data (40 features in total), and the postorder incorporates online media data (30 features in total).

In the “preorder + postorder” data correspondence method, the “linear + nonlinear” method is BSEE1, and the “nonlinear + linear” method is BSEE2. In the comparison of various data inclusion methods, the regression prediction performance of BSEE1 is significantly better than that of BSEE2, and the prediction effect is shown in Table 4.

**Table 4:** Comparison of regression effects of two kinds of BSEE.

Model	Dataset	MSE	MAE	RMSE	*R*^2^
BSEE1	Training	12.9075	2.6348	3.5927	0.9478
	Testing	142.1827	7.6052	11.9240	0.6266
BSEE2	Training	17.1359	3.0291	4.1396	0.8969
	Testing	20.2084	3.3688	4.4954	0.8698

*BSEE* bootstrap-sampling synchronous evolutionary ensemble. *MSE* mean-square error, *MAE* mean absolute error, *RMSE* root-mean-square error, *R*^2^ R-square.

**Fig. 5:**
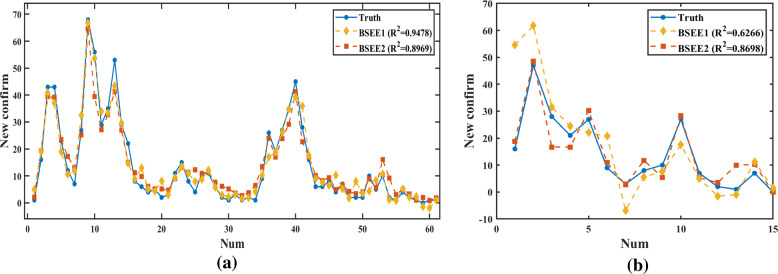
Comparison of regression effects of two kinds of BSEE. **a** Training set, **b** test set.

**Fig. 6:**
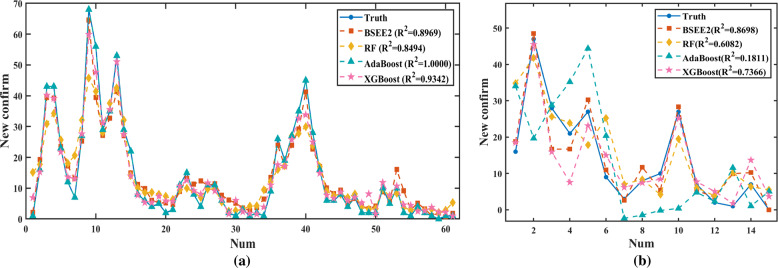
Comparison of regression effects of various methods. **a** Training set, **b** test set.

In the comparison of various methods, the regression performance of BSEE2 is significantly better than that of traditional ensemble methods, and the *R*^2^ value reaches 0.8698, as shown in Table 5. The fitting curve of the regression effect evaluation also confirms this conclusion, as shown in Figs. 5 and 6. Hence, BSEE2 has the best prediction accuracy on the test set, and our improved ensemble method can predict the development trend with better accuracy than traditional methods.

**Table 5:** Comparison of regression effects of various methods.

Model	Data	MSE	MAE	RMSE	*R*^2^
BSEE2	Training	17.1359	3.0291	4.1396	0.8969
	Testing	20.2084	3.3688	4.4954	0.8698
RF	Training	37.2572	4.1326	6.1039	0.8494
	Testing	64.7970	6.0360	8.0497	0.6082
AdaBoost	Training	<0.0001	<0.0001	<0.0001	1.0000
	Testing	187.5624	10.9839	13.6953	0.1811
XGBoost	Training	11.9012	2.4786	3.4498	0.9342
	Testing	31.3187	4.0827	8.3788	0.7366

### Generalization of the early warning model

**Fig. 7:**
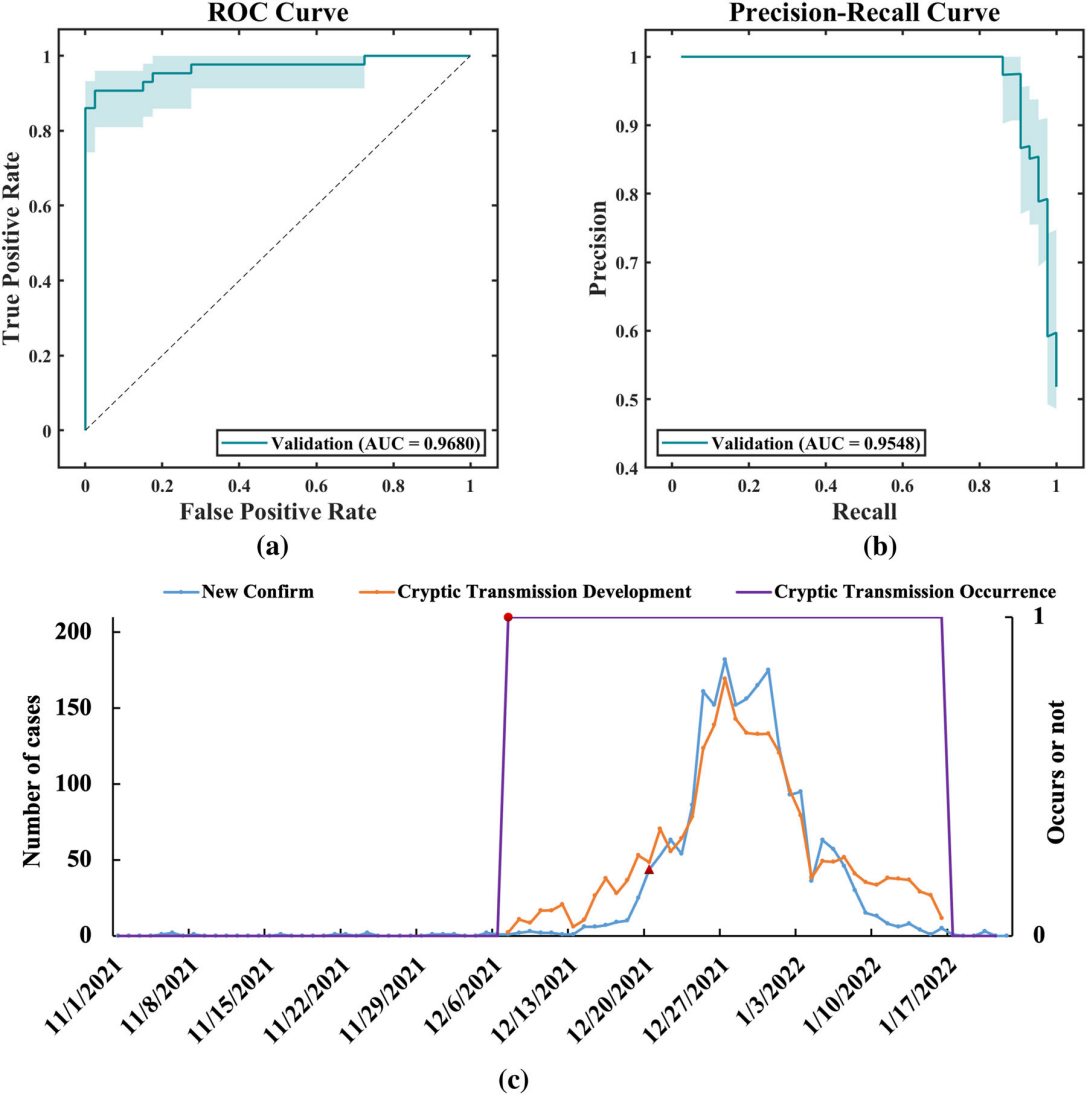
Occurrence, development, and fitting of the external validation data. **a** The ROC curves of early warning; **b** the PR curves of early warning; **c** the fitting curve of the occurrence and development of the epidemic. The blue line represents the actual number of confirmed cases per day, the purple line represents the predicted occurrence result of the model, the orange line represents the predicted development result of the model, the triangular icon represents the time point of the official report of cryptic transmission, the circular icon represents the time point of our prediction of the occurrence of cryptic transmission, and our prediction time is 13 days ahead of the official date.

We selected the best classification and regression model, and using the Shaanxi Province data as the external verification dataset, we carried out the generalization analysis of the occurrence and development of the early warning of cryptic transmission. See Fig. 7 for the fitting curve. The results of external verification show that our early warning model successfully found cryptic transmission on December 7, 2021. According to official sources, cryptic transmission was not discovered by the traditional monitoring system until December 20. Hence, our early warning model had better foresight.

The PRE, SEN, SPE, ACC, F1, ROC-AUC and PR-AUC values of USEE3 were 0.8367, 0.9535, 0.8000, 0.8795, 0.8913, 0.9680, and 0.9548, respectively, in the cryptic transmission occurrence warning. The MSE, MAE, RMSE, and *R*^2^ values of BSEE2 were 358.9284, 15.5131, 18.9549, and 0.8255, respectively, in the developmental warning of cryptic transmission. Hence, in terms of external verification data, our early warning model can also correctly predict the trend of cryptic transmission. Our model can be applied to early warning systems in other regions without further training, and it has good generalization ability.

## Discussion

During the occurrence and development of the COVID-19 pandemic, the impact of cryptic transmission could not be ignored. Since 2020, most countries have been affected by the cryptic transmission of the pandemic, mainly because they did not recognize the importance of cryptic transmission and did not take targeted prevention and control measures^7^. After 2020, there was no nationwide epidemic of COVID-19 in China, but there were still frequent regional occurrences. The reason is that the existing prevention and control measures do not pay enough attention to cryptic transmission. In previous studies, there were few related studies on cryptic transmission, and the scope of concern was small, and no reports focused on the cryptic transmission of COVID-19 in China have been published thus far. China’s total population ranks first in the world, and the prevention and control of epidemic outbreaks are relatively difficult, and the consequences are often more serious. Hence, it is of great significance to focus on the latent spread of COVID-19 in China.

Previous similar studies only focused on Internet retrieval data, and the utilization of other Internet big data was low^13–16^. Hence, in the application of Internet big data, we concentrated on two important aspects of comprehensiveness and relativity. (1) Comprehensiveness: Traditional research on Internet data often only focuses on retrieving data, such as a series of studies represented by the GFT, but the Internet retrieval data cannot fully represent the Internet behaviour of the population, so there is a problem of generalization. Hence, we include population migration data and Internet media data to reflect Internet behaviour as comprehensively as possible. In the comparative study of various datasets, we find that when all types of Internet datasets are comprehensively used, the best prediction effect can be obtained. (2) Relativity: Although big data on the internet can reflect the internet behaviour of people within a certain period, regional internet retrieval data are greatly affected by national data, and the direct application of regional data may not explain cases of spontaneous retrieval. As a result, our approach is to determine the ratio of regional to national data, thereby improving the accuracy of the analysis and eliminating abnormal errors in the retrieved data.

At present, the cryptic transmission situation is complex. We should not only monitor it early in the occurrence stage but also study and evaluate it correctly during the development stage. Based on the profound significance and high difficulty of COVID-19 cryptic transmission early warning, we collected multisource Internet big data and improved the research methods. We also proposed an improved evolutionary algorithm (ICHIO) and implemented the “sampling-feature-hyperparameter-weight” synchronous optimization strategy under its guidance, which improves the efficiency of feature subset selection and hyperparameter optimization. In occurrence warning, we proposed an USEE, and in the development warning, we proposed a BSEE. Based on the above methods, we devised an early warning protocol for the occurrence and development of COVID-19 cryptic transmission. First, the classification method was used to judge whether there was cryptic transmission, and then the regression method was used to fit the development trend of cryptic transmission. The processing method of “first occurrence and then development” can effectively prevent the data noise caused by cryptic propagation and emission and is conducive to achieving high prediction accuracy.

We conducted time series analysis on the data of the outbreak stage, deeply examined the epidemic characteristics of the epidemic, and confirmed the feasibility of using Internet big data to provide an early warning for COVID-19. We classified the in-depth mining of Internet retrieval data, population migration data and Internet media data to dynamically monitor the incidence. In the early warning of the occurrence and development of cryptic transmission, we used the concept of synchronous evolution and integration to model. In the warning of the occurrence, we constructed USEE, and when incorporating data from the three sources, the model had the best predictive accuracy on the test set. In the warning of the development, we construct BSEE, and the model has the best prediction accuracy on the test set when the “nonlinear + linear” order is adopted. On the external validation data, the optimal model successfully predicted the occurrence and development trend of cryptic transmission, and our early warning was 13 days earlier than the official warning, which demonstrated a good advantage. Hence, our method not only has good prediction accuracy but also has strong generalization ability and can be flexibly used in the prediction of cryptic transmission in various regions. Our method can effectively overcome the lag effect existing in traditional monitoring models and effectively save monitoring costs. It has great economic benefits and scientific value. This research strategy integrates a variety of early warning tasks, is based on a number of Internet big data sources, and combines the two with a variety of models. It is an extension of research in the field of traditional infectious disease monitoring and has important practical significance and theoretical innovation value.

The smooth implementation of synchronous optimization relies on the guidance of the improved evolutionary algorithm (ICHIO). Twenty-three standard test functions show that the ICHIO algorithm is conducive to improving the feature selection ability and has good stability. The ICHIO algorithm can obtain better global search performance and avoid too fast of a convergence and falling into a local optimal solution. Compared with previous optimization methods, our improvement is that we do not simply use fixed values for feature selection but creatively put forward the concept of “feature scale”. The initial value of the feature scale was randomly generated. After that, we carried out iterative optimization to update and optimize using the ICHIO algorithm and continuously approach the optimal value. The proposed feature scale is conducive to the efficient discovery of key features and the elimination of redundant features to improve the operation efficiency and model accuracy. The whole process of synchronous optimization makes full use of ICHIO’s global optimization ability and optimization efficiency and effectively enhances the meta-learning ability of ensemble learning.

Although the design of this study is reasonable and strictly implemented, there is some room for improvement. We look forward to the following future research directions. (1) In the prediction of the development of the cryptic transmission of external data, there is still some room for improvement. Hence, in practical applications, it is necessary to build an accurate prediction model according to the characteristics of each region. (2) In terms of data sources, we only selected three kinds of Internet big data. In the future, we will carry out multidimensional and wide-ranging analyses from the aspects of spatial distribution, population distribution, and pathogenic factors to better develop an early warning. (3) Any single algorithm has certain limitations. In future work, researchers can consider combining the ICHIO algorithm with other meta heuristic algorithms to improve its optimization ability.

To warn of the occurrence and development of COVID-19 cryptic transmission, we propose an early warning method using Internet multisource big data and adopting a comprehensive and relative strategy. We also innovatively propose the ICHIO algorithm and use “sampling-feature-hyperparameter-weight” synchronous optimization strategies to improve the computational performance and efficiency of the early warning model. Our model has achieved good early warning efficiency on both internal training data and external verification data. We believe that our research will improve the ability to perceive an epidemic in the early stage and make a significant contribution to the early warning of COVID-19. Our method can be extended to future predictions in other regions and has important practical significance and innovative theoretical value.

## Methods

### Data acquisition

Ethical approval was not required for the analysis presented in this paper. Data was obtained in an anonymised and aggregated format. The data we selected included diagnosis data and Internet big data, which can be further divided into Internet retrieval data, population migration data, and Internet media data.

1. Diagnosis data

Among the national data, the data of daily confirmed cases of COVID-19 came from official channels such as the national health commission and the health commissions of various regions (including 31 provinces and cities, excluding Hong Kong, Macao, and Taiwan). The study period was from January 21 to March 1, 2020, during the COVID-19 outbreak stage.

Regarding regional data, the data of Heilongjiang Province was used as the internal training data, and the data of Shaanxi Province was used as the external verification data. In the occurrence warning (classification), the data span of Heilongjiang Province was 340 days and that of Shaanxi Province was 83 days. After screening out the time of occult transmission by classification, the development of early warning (regression) research continued. The data span of Heilongjiang Province was 76 days and that of Shaanxi Province was 43 days. The time span of each dataset for each task is shown in Table 6.

**Table 6:** Time span of each dataset for each task.

Dataset	Usage	Time span	Days
Internal data	Warning of occurrence	2021.1.10–2021.12.15.	340
	Warning of development	2021.1.11–2021.2.5, 2021.9.20–2021.10.5, 2021.10.27–2021.11.15, 2021.12.1–2021.12.14.	76
External data	Warning of occurrence	2021.11.1–2022.1.22.	83
	Warning of development	2021.12.5–2022.1.16.	43

Both Heilongjiang Province and Shaanxi Province belong to the “thousand-case level” of local outbreaks. The transmission chain was difficult to track, and local authorities failed to achieve short-term control. Both provinces were characterized by large scale, frequent incidents and typical distribution. Hence, the data of these two provinces have high research value.

2. Internet big data

Baidu searches account for 84.27% of China’s search engine market share (as of March 2022), making Baidu the number one search engine in China (https://gs.statcounter.com). TikTok’s daily active volume on China’s mobile internet will reach 600 million in 2021 and 800 million in 2022 (http://www.cac.gov.cn). Furthermore, the popularity of social media has influenced the stock market. In general, the Baidu search index, Baidu migration data, and Baidu information index represent China’s traditional internet, the TikTok composite index represents China’s mobile internet, and the stock index is an important indicator for measuring the impact of social media on China’s economy and society. As a result, given China’s national characteristics and market demand, we chose the above data as being representative of internet big data.

(1) Internet search data: We selected the Baidu search index as the representative of Internet search data. The Baidu index is a sharing platform based on a large amount of user retrieval behaviour data. It uses the keyword retrieval volume as a statistical object and uses a system algorithm to perform a weighted summation of the number of retrievals of each keyword in the search engine. We collected a total of 33 keywords in 7 categories according to the characteristics of COVID-19, as shown in Table 7.

**Table 7:** Keyword list of the Baidu search index.

Type	Associated words and phrase	Number
Name	COVID-19/2019-nCoV/SARS-cov-2/ corona virus disease 2019/coronavirus disease 2019	5
Symptom	symptoms of COVID-19/new crown symptoms/cold/headache/body temperature/fever/respiratory tract infections	7
Prevention	mouthpieces/thermometer/disinfectant/epidemic prevention and control/new crown vaccine/epidemic prevention and control measures	6
Diagnosis	nucleic acid test/new crown virus/coronavirus/new coronavirus/new coronavirus mutations/RNA virus/CT	7
Therapy	antipyretics/coldrex/antiviral drugs/LianHua QingWen granule/Jinhua Qinggan granule/Huashi Baidu granule/Xuebijing	7
Related diseases	SARS/influenza/atypical pneumonia	3
Other	COVID-19 epidemic/COVID-19 latest news/coronavirus disease 2019 latest news	3

*COVID-19* corona virus disease 2019, *SARS* severe acute respiratory syndrome, *RNA* ribonucleic acid, *CT* computed tomography.

(2) Population migration data: We selected Baidu migration data as the representative population for migration data. Baidu migration data are important migration data obtained from Baidu Maps, including the migration scale index and the migration scale index of each region.

(3) Online media data: We selected the Baidu information index, TikTok index and stock index as representatives of online media data, as shown in Table 8. The Baidu information index is based on intelligently recommended content, showing the degree of attention and changing trends in news information. The TikTok composite index measures the synthesized sound volume of specific keywords and is obtained by weighting the relevant internal capacity, user views, searches and other behavioural data. The stock index refers to the value compiled by financial institutions representing changes in the stock market, which can be used to predict social, political, and economic developments^40^.

**Table 8:** Keyword list for online media data.

Type	Keywords
Baidu information index	COVID-19, coronavirus, epidemic, infectious disease, pneumonia, SARS, influenza, vaccine
TikTok composite index	COVID-19, coronavirus, epidemic, infectious disease, pneumonia, SARS, influenza, vaccine
Stock sector index	Flu, biomedical, pharmaceutical ETF, pharmaceutical business, vaccine

*ETF* exchange traded fund.

### Characteristics and processing of Internet big data

**Fig. 8:**
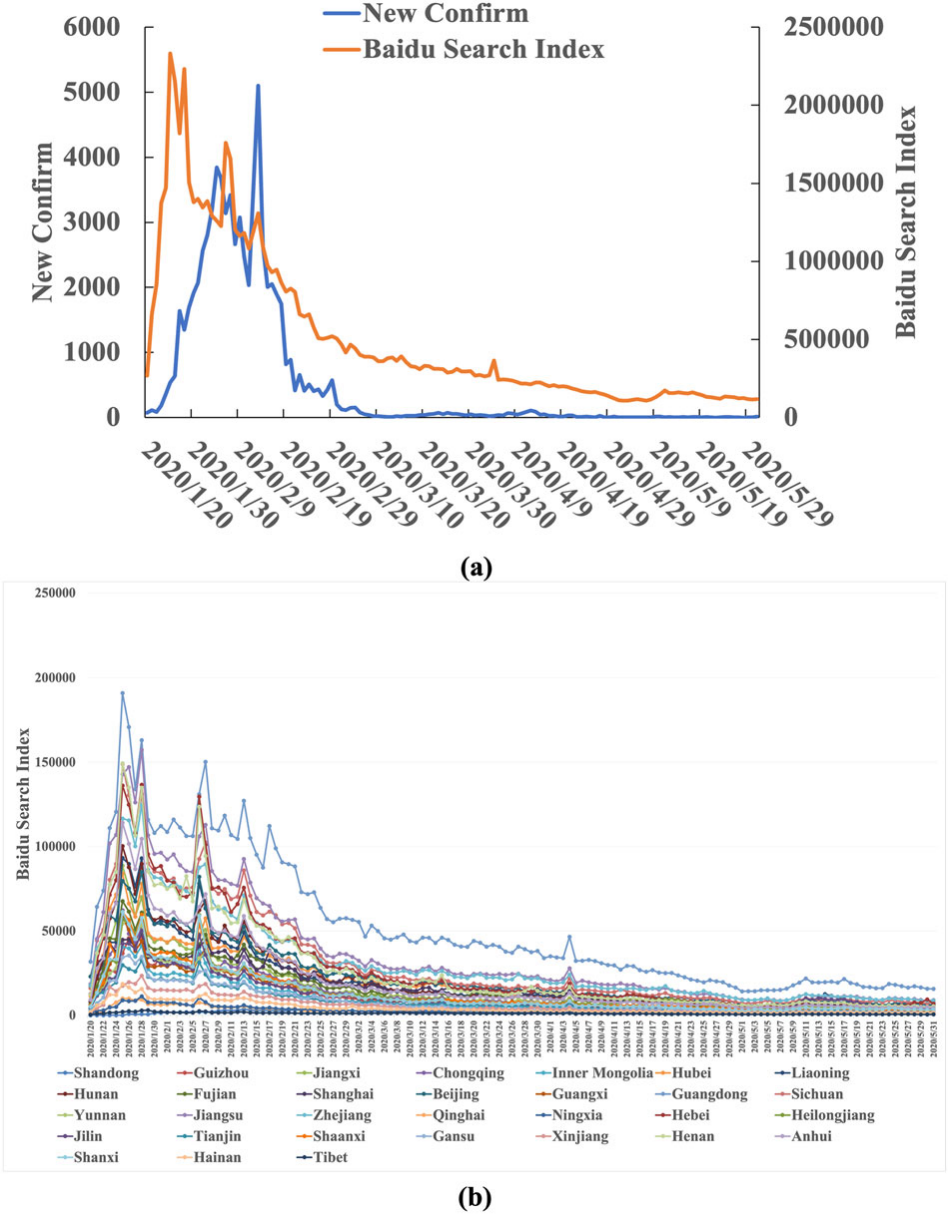
Baidu index and time series distribution of daily new cases. **a** Shows the overall situation of the whole country, and **b** shows the situation of each region.

In the outbreak stage, we analyzed the relationship between the temporal characteristics of the epidemic data and the Baidu search index and confirmed the scientific nature of the Internet retrieval data in predicting the epidemic. We included the subject words directly related to the epidemic and analyzed the temporal distribution characteristics of the Baidu index. Figure 8 shows the time trend of the national and regional Baidu index and newly confirmed cases. The results show that the national Baidu search index is correlated with the epidemic trend in terms of temporal characteristics and can be used as an index for early warning of epidemics.

**Fig. 9:**
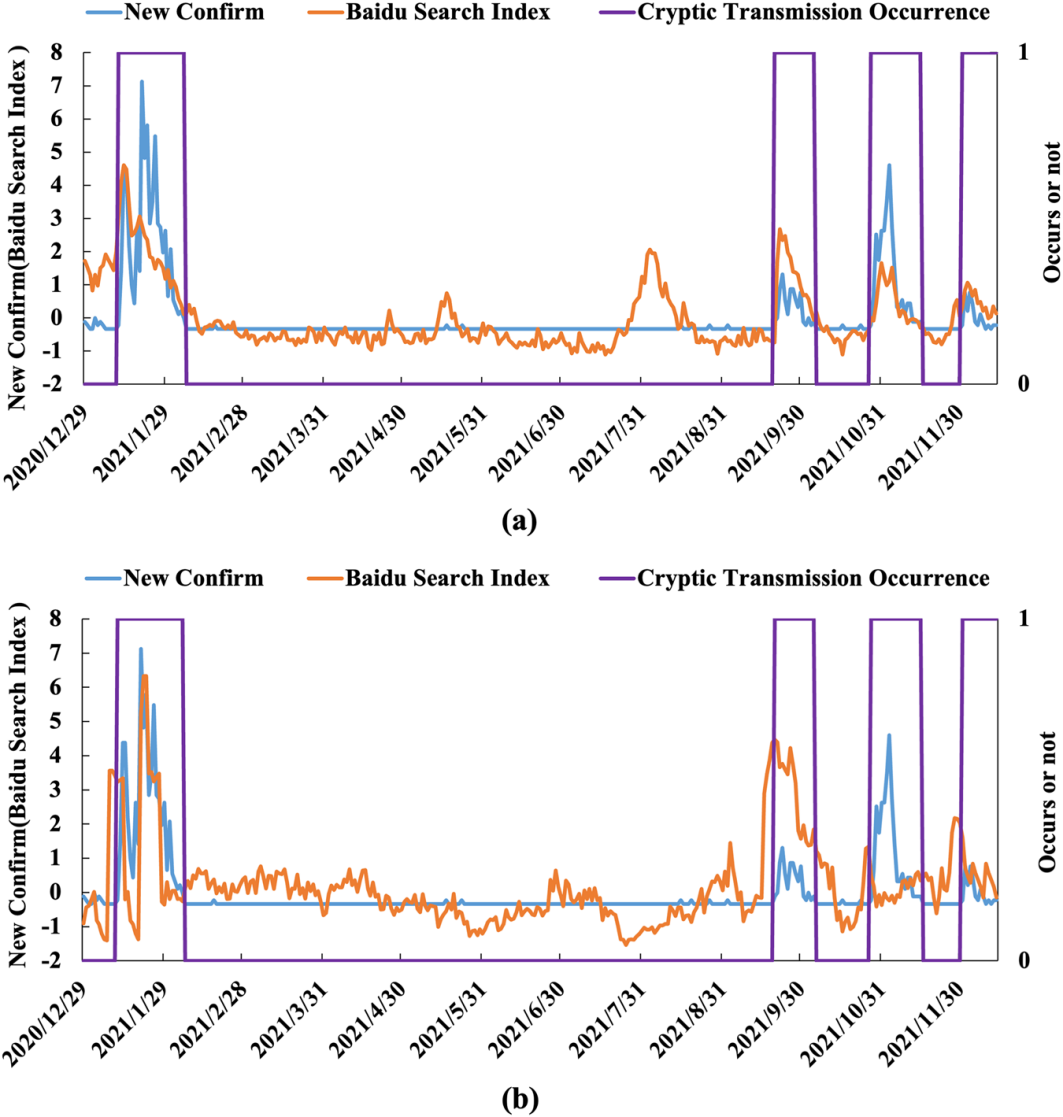
The relationship between Internet retrieval data and the occurrence and development of cryptic transmission. The relationship between the retrieval data and the occurrence and development of cryptic transmission is shown. **a** Shows national data, and **b** shows a ratio (regional/national data) that expresses the concept of “relativity”. The left vertical axis represents the confirmed data and Baidu search index (after standardized processing), and the right vertical axis represents whether hidden transmission occurs (0 represents no occurrence and 1 represents occurrence). Regional Internet retrieval data are easily affected by the overall level of the country and Internet media information, so the proportion of regions in the country is used to construct the dataset.

First, we consider Internet retrieval data (Heilongjiang Province) as an example of data preprocessing and analyze the availability of big data on the Internet. The correlation analysis between regional Internet retrieval data and epidemic diagnosis data is shown in Fig. 9a. From July to August 2021, although there was no cryptic transmission of COVID-19, there was an abnormal peak in Internet search data on the topic. The national COVID-19 epidemic has influenced regional retrieval data, and using only regional network retrieval data may affect the accuracy of the predicted results. To avoid the impact of the epidemic in other regions of the country on local internet data, we adjusted the data to include only regional/national internet retrieval data. That is, we compute the ratio of regional network retrieval data to national network retrieval data to correct the data accuracy using the idea of “relativity”. According to the corrected results, there were obvious antecedent characteristics in the internet retrieval data, and there were no other abnormal peaks. See Fig. 9b for details. We implemented Z score standardization for all data.

### Improved algorithm

Traditional optimization algorithms often suffer from slow convergence and a tendency to fall into local optimality. Hence, we propose an improved metaheuristic algorithm with the advantages of accurate solution method and robust computational power, aiming to implement optimization efficiently and accurately.

**Fig. 10:**
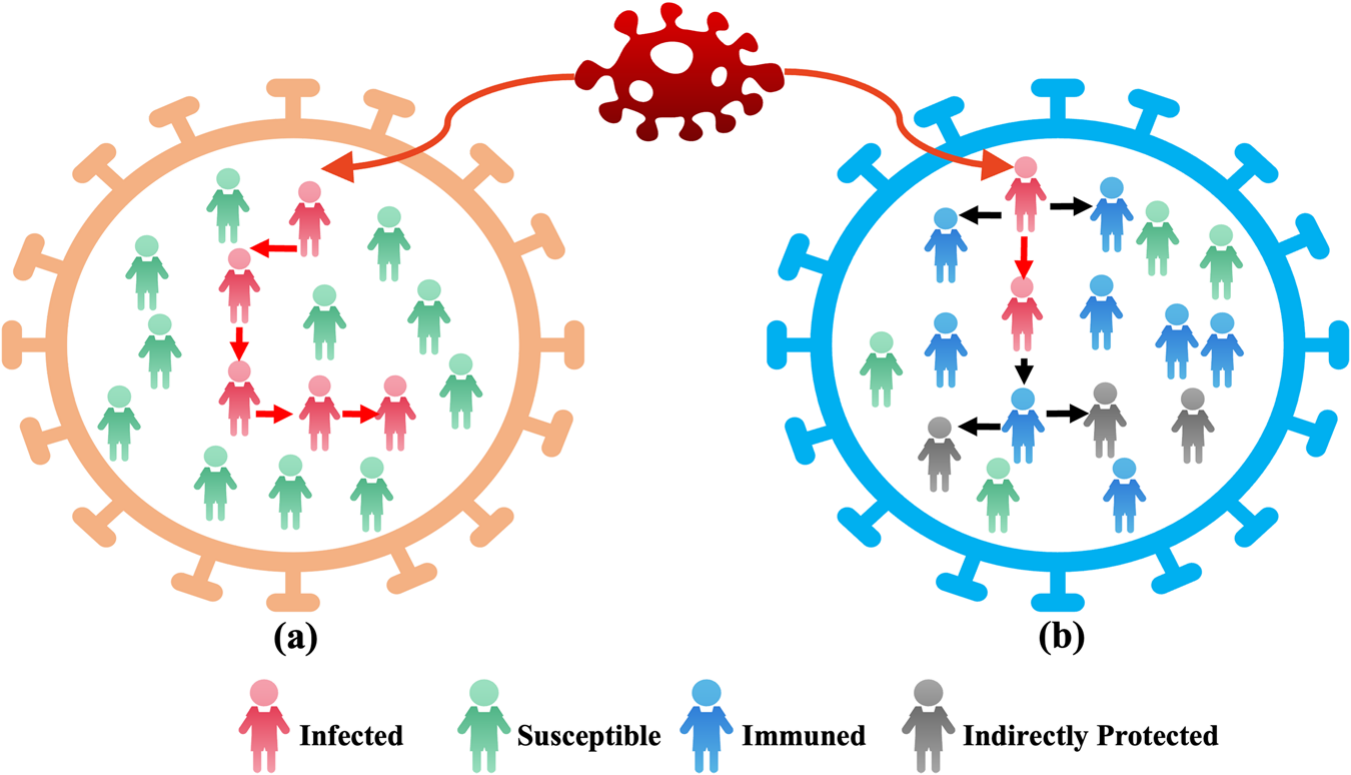
Population immunity simulation in the CHIO model. Red represents infection cases, green represents susceptible cases, blue represents immune individuals, and grey represents indirectly protected cases. **a** This group had no immune individuals or isolated infection cases and did not increase social distance, which eventually led to case transmission. **b** This group has immune individuals and protective barriers, so it can effectively prevent the spread of the virus.

In 2020, inspired by the social distance and swarm immune strategy, the coronavirus herd immune optimizer (CHIO) algorithm was proposed as a human-based optimization algorithm^37^. When the proportion of individuals having immunity gradually increases and reaches a state of group immunity, it can better protect susceptible individuals, as shown in Fig. 10.

**Fig. 11:**
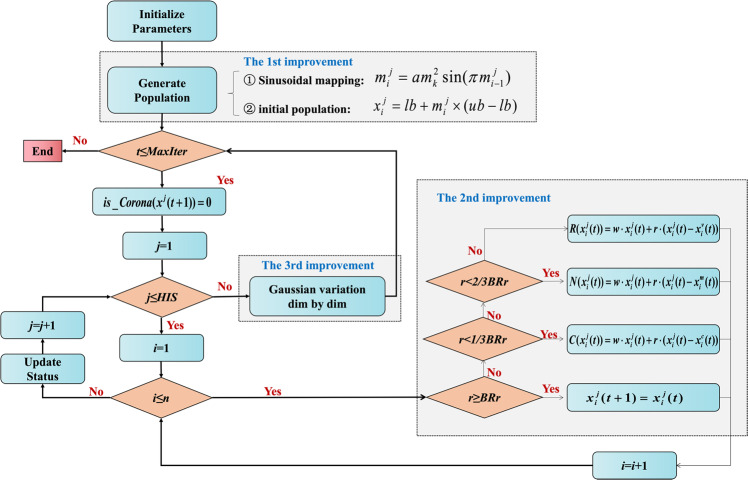
Principle of the ICHIO algorithm. The figure shows the order of operations of ICHIO, where the shaded box corresponds to the improvement compared to the baseline method.

The CHIO algorithm has slow convergence speed and easily falls into local optimums. Hence, based on the CHIO algorithm, we propose an improved coronavirus herd immunity optimizer (ICHIO) algorithm, which uses strategies such as sinusoidal chaotic mapping, inertia weight factor and dimensional Gaussian mutation to improve the ability of the algorithm. The principle of the ICHIO is illustrated in Fig. 11.

First, we determine the objective function $f(x)$ of the problem to be solved and the initialized population $U$.

The objective function can be expressed by the following formula:

1 $$\min f(x),x \in [lb,ub]$$

where $ub$ and $lb$ represent the upper and lower bounds of the search space, respectively.

$U$ is stored in the form of a two-dimensional matrix. The rows of matrix $U$ represent individuals (the number of whom is $HIS$), and the columns of matrix $U$ represent the problem to be solved (the dimension of which is $n$), which can be expressed as:

2 $$U = \left[ {\begin{array}{*{20}{l}} {x_1^1,} \hfill & {x_2^1,} \hfill & { \cdots ,} \hfill & {x_n^1} \hfill \\ {x_1^2,} \hfill & {x_2^2,} \hfill & { \cdots ,} \hfill & {x_n^2} \hfill \\ \cdots \hfill & \cdots \hfill & { \cdots ,} \hfill & \cdots \hfill \\ {x_1^{HIS},} \hfill & {x_2^{HIS},} \hfill & { \cdots ,} \hfill & {x_n^{HIS}} \hfill \end{array}} \right]$$

To overcome the blindness in the previous initialization, the sinusoidal chaotic mapping strategy is used to initialize the population, and $x_i^j$ is used to represent the *i*-th-dimension value of the *j*-th individual in population $U$, which is defined as follows:

3 $$x_i^j = lb + m_i^j \times (ub - lb)$$

where $m_i^j$ is generated using the sinusoidal chaotic mapping strategy and can be expressed as:

4 $$m_i^j = a(m_{i - 1}^j)^2\sin (\pi m_{i - 1}^j)$$

The initial value of $m_0^j$ for each individual in the population is 0.7, and the value of *a* is 2.3. Sinusoidal mapping is distributed in [0, 1], which uses chaos instead of random initialization to more evenly distribute the population in the search space.

Second, the inertia weight factor $w$ can adaptively improve the convergence speed of the algorithm, which can be defined as follows:

5 $$w = 1 - (t/{\rm{MaxIter}})^2$$

here, ${\rm{MaxIter}}$ represents the maximum number of iterations, and *t* represents the current iteration number.

We substitute $w$ into the original algorithm, and $C(x_i^j(t))$, $N(x_i^j(t))$ and $R(x_i^j(t))$ represent the influence of social distancing on infected cases, susceptible cases and immune cases, respectively, which can be expressed as:

6 $$C(x_i^j(t)) = w \times x_i^j(t) + r \times (x_i^j(t) - x_i^c(t))$$

7 $$N(x_i^j(t)) = w \times x_i^j(t) + r \times (x_i^j(t) - x_i^m(t))$$

8 $$R(x_i^j(t)) = w \times x_i^j(t) + r \times (x_i^j(t) - x_i^v(t))$$

*t* represents the current iteration number, and $x_i^c(t)$, $x_i^m(t)$, and $x_i^v(t)$ represent the *i*-th-dimension values of the current infected cases, susceptible cases, and immune cases.

Subsequently, each iteration performs dimension-wise Gaussian mutation to improve the performance of global search optimization, which can be expressed as:

9 $$X{bestnew}(i) = w \times X{best}(i) + {randn} \times X{best}(i)$$

here, $w$ represents the inertia weight factor, $X{best}(i)$ represents the best value of the $i$ dimension of the best individual in the current population, $X{bestnew}(i)$ represents the value of $X{best}(i)$ after dimensional Gaussian variation, and ${\rm{randn}}$ represents a random number conforming to a normal distribution.

Finally, the greedy strategy is used to update the optimal solution, which is expressed as:

10 $$X{best} = \left\{ \begin{array}{l}X{bestnew},{\rm{if}}\,{{{\mathrm{ }}}}f(X{bestnew}) \,<\, f(X{best})\\ X{best},{else}\end{array} \right.$$

**Fig. 12:**
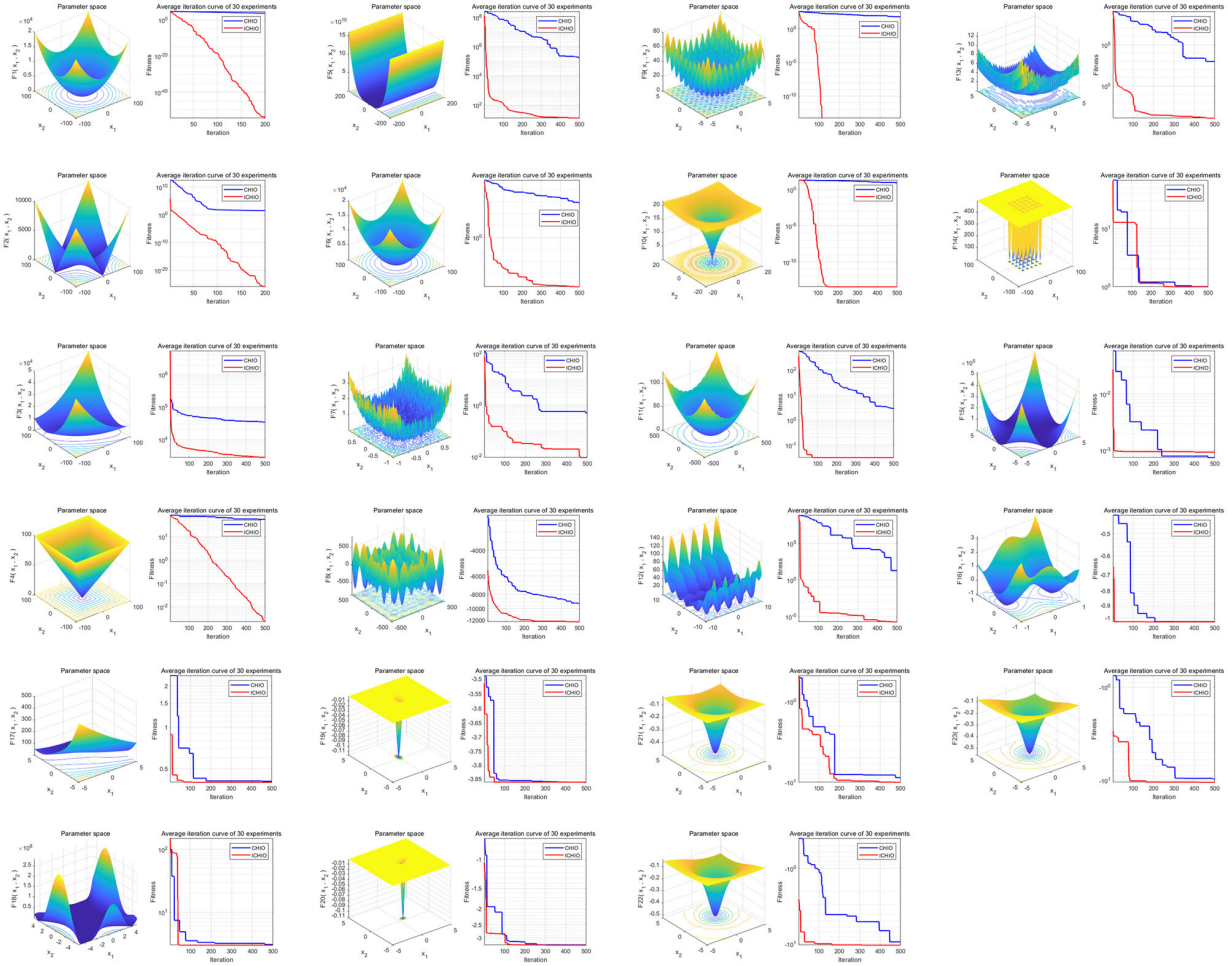
Functions and convergence plots. When using the ICHIO algorithm, the three-dimensional surface diagram in the figure shows the two-dimensional search space of each benchmark function. The line chart shows the convergence trend of the first solution of the first dimension of each benchmark function and compares the trends of the CHIO and ICHIO algorithms.

We evaluate the evolutionary algorithm’s performance using 23 common test functions: compared to those of the baseline algorithm, the overall convergence speed and global optimization ability of ICHIO are significantly improved (see Fig. 12 for details). As a result, the ICHIO algorithm performs admirably and is used as the guiding algorithm in the “Synchronization optimization” section below.

### Synchronous optimization

**Fig. 13:**
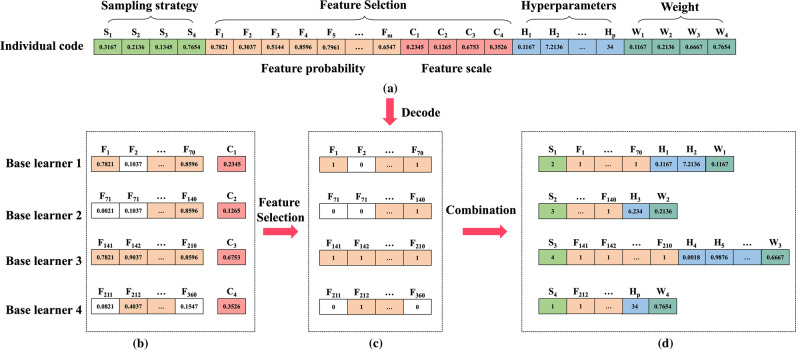
“Sampling-feature-hyperparameters-weights” simultaneous optimization process. **a** ICHIO consists of a dataset sampling strategy, feature probabilities, feature scales, hyperparameters and weights. **b** Comparison of feature probabilities (*F*_m_) and feature scales (*C*_c_) for each base learner with feature subsets selected depending on the size of the relationship. **c** The result of feature selection. **d** Combination of “sampling-feature-hyperparameter-weight” and simultaneous optimization to obtain the optimal individual value after several iterations of the objective function.

The ICHIO algorithm enhances the meta-learning ability of the ensemble model and performs steps of dataset sampling, feature selection, hyperparameters optimization, and determination of the weights of the base learners synchronously. The synchronous optimization process is shown in Fig. 13.

First, the evolutionary algorithm is initialized. The individuals in the group all use the m-dimensional real number corresponding to the dataset sampling strategy. The *n*-dimensional real number corresponds to the feature subset selection, the o-dimensional real number corresponds to the feature scale, the p-dimensional real number corresponds to the hyperparameter selection, and the *q*-dimensional real number corresponds to the weights of the base learners. When initially constructing the optimization model, the ICHIO algorithm consisted of five parts: dataset sampling strategy (*S*_m_), feature probability (*F*_n_), feature scale (*C*_o_), hyperparameter (*H*_p_), and base learner weight (*W*_q_). During initialization, we used a chaotic strategy to set random numbers within a specific range for each individual.

The feature selection step depends on the implementation of feature probability and feature scale and compares the relationship between the two. When the feature probability is higher than the feature scale, the feature is selected (the recorded value is 1). When the feature probability is lower than the feature scale, the feature is excluded (the recorded value is 0). The features are then recombined with the value of 1 in a feature subset. We then determine the corresponding feature probability and feature scale combination for each base learner in the integrated model, the sampling dataset corresponding to each base learner according to the size ranking relationship of *N* real numbers in *S*_n_, and determine *H*_p_ and *W*_q_ at the same time.

The initial population is substituted into the objective function of the classification or regression task, and the individual corresponding to the optimal value is sought, which is the optimal solution obtained in the first iteration. This step is then repeated using the ICHIO update strategy until the best individual in the population meets the need or the maximum number of iterations is reached, at which point the combination of “sampling strategy + feature subset + hyperparameters + weights” is the desired result. The above “sampling-feature-hyperparameters-weights” optimization strategy allows various base learners to learn the diversity of data, enhance the variability and diversity of models, and achieve the best combination of the base learners’ own weights, features, datasets, and hyperparameters.

### Classification ensemble model

In the classification task, the onset time accounted for a small proportion of the total time, and there was data imbalance. Hence, we combined undersampling and integration methods, and the two methods complemented each other. The advantage of random undersampling (RUS) is that it solves the problem of data imbalance, increases the accuracy of model training, reduces the burden of model training, and improves the efficiency of model operation. The advantage of the ensemble method is that it prevents the problem of information loss caused by undersampling.

**Fig. 14:**
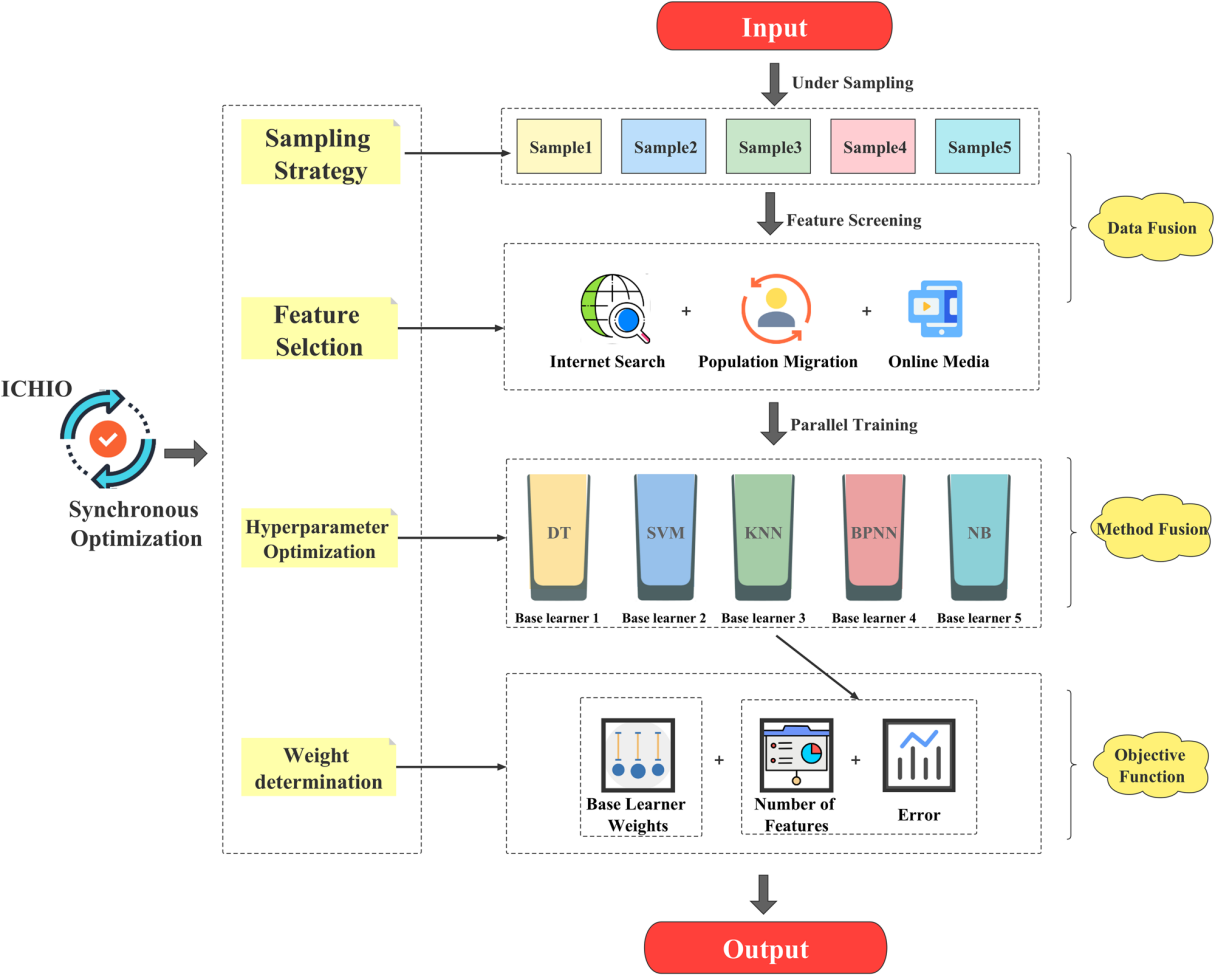
The structure of the USEE model. DT Decision Tree, SVM Support Vector Machine, KNN K-Nearest Neighbour, BPNN BP Neural Network, NB Naive Bayes.

The base learners corresponded to the undersampled datasets, and each base learner had its own weight. We performed a “sampling-feature-hyperparameters-weights” synchronous optimization strategy to construct an USEE model. The structure of the model is shown in Fig. 14.

The specific steps of USEE construction are as follows.

(1) Dataset sampling: The training set and the test set are distinguished at a ratio of 3:1, and 10-fold cross-validation is used in the training process. Five balanced datasets were obtained after undersampling the training set five times, and ICHIO was used to guide the datasets input by each base learner.

(2) Feature selection and hyperparameter optimization: Under the guidance of ICHIO, features, and hyperparameters are optimized simultaneously.

(3) Determination of weights: The adaptive weight of the base learner is composed of the baseline weight and the correct rate. Taking *w*_*1*_~*w*_*5*_ to represent the baseline weight and *e*_*1*_*~e*_*2*_ to represent the correct rate, the *i*th base learner weight *W*_*i*_ can be expressed as:

11 $${{{\mathrm{W}}}}_i = w_i \times e_i$$

(4) Ensemble modelling: Using ICHIO coding, the probability scores of each base learner are weighted and combined to form an integrated model. Finally, the classification probability score Q output by the model can be expressed as:

12 $$Q = \mathop {\sum}\limits_{i = 1}^5 {W{}_i \times P_i}$$

here, *P*_*i*_ represents the posterior probability score output by the *i*th base learner, and the corresponding classification prediction result is obtained according to the probability score P. Predict the validation set (10%) according to the integrated model, perform 10-fold cross-validation, and use the 10-fold validation set prediction results and the real results to calculate the comprehensive error value.

(5) Objective function: The objective function is composed of error, L2 regularization of weight, and L1 regularization of feature quantity. The introduction of the regular term is aimed at avoiding the risk of overfitting. Due to the data imbalance in the original sample, we focused on the detection rate of hidden propagation and used the (1-F1 index) as the error term in the objective function, which can be expressed as follows:

13 $$\min \left( {1 - F_1} \right){{{\mathrm{ + }}}}\lambda \mathop {\sum}\limits_{i = 1}^{{{\mathrm{5}}}} {W_i^2} + \gamma \mathop {\sum}\limits_{i = 1}^{{{\mathrm{5}}}} {N_i}$$

Among them, *F*1 represents the *F*1 index, *Ni* represents the number of final training features of the *i*th base learner, and *λ* and *γ* are hyperparameters, which are set according to the data dimension (here, *λ* = 0.01, *γ* = 0.001).

### Regression ensemble model

In the regression task, limited by the small onset time, there was insufficient sample size, which leads to serious overfitting. Thus, we combined ensemble learning with bootstrap sampling. Bootstrap sampling is a kind of sampling with replacement, which belongs to the important data processing strategy of small sample learning. We trained various base learners in parallel by sampling the dataset multiple times and combining the weights of base learners to obtain predicted results.

**Fig. 15:**
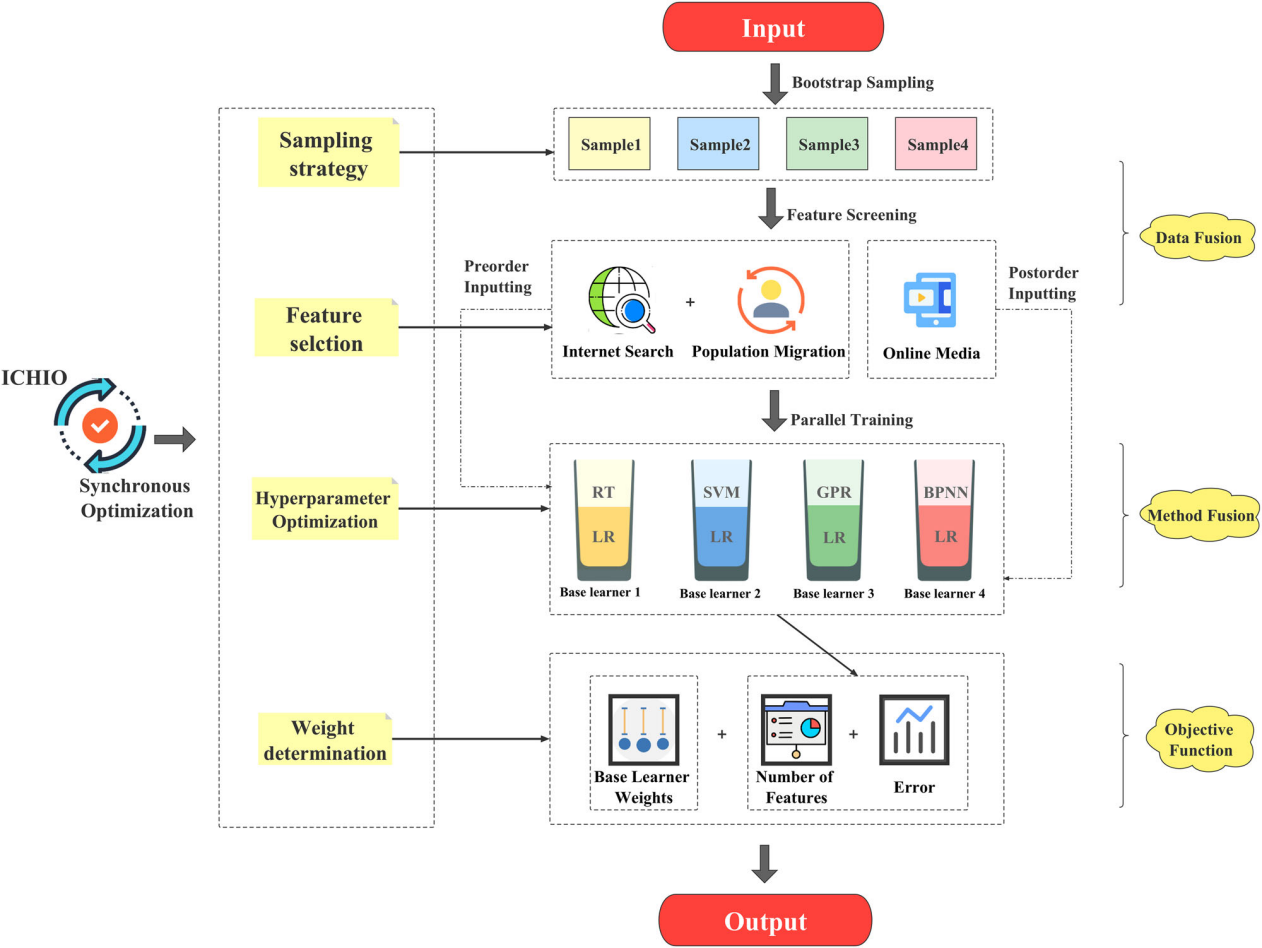
The structure of the BSEE model. RT Regression Tree; SVM Support Vector Machine; GPR Gaussian Process Regression; BPNN BP Neural Network; MLSR Multiple Linear Stepwise Regression.

In the study of ensemble learning of traditional regression tasks, the base learner is often a single linear or nonlinear method, which has a limited effect on feature fitting. In view of the multisource network big data collected in this study, we propose the concept of an “integrated base learner”; each base learner is composed of a preorder and a postorder, and each of these input data from various sources. Linear methods are suitable for processing stationary data, while nonlinear methods are suitable for processing high-dimensional, noisy data. The integration is carried out by means of weighted summation, and each base learner adaptively incorporates features. Hence, we propose a BSEE model, which uses ICHIO to guide the synchronous optimization of “sampling-feature-hyperparameters-weights”. The structure of the model is shown in Fig. 15.

The specific steps of BSEE construction are shown below.

(1) Dataset sampling: The training set and the test set are distinguished at a ratio of 4:1, and 10-fold cross-validation is used in the training process. Four datasets were obtained after 5 self-samplings of the training set, and ICHIO was used to guide the datasets included in each base learner.

(2) Feature selection and hyperparameter optimization: Under the guidance of ICHIO, features and hyperparameters are optimized simultaneously.

(3) Determination of weights: In the optimization of weights, the weights *w*_*1*_~*w*_*4*_ of each base learner are determined and normalized to *W*_*1*_~*W*_*4*_. The *i*th base learner weight Wi can be expressed as follows:

14 $${{{\mathrm{W}}}}_i = \frac{{w_i}}{{\mathop {\sum}\nolimits_{i = 1}^{{{\mathrm{4}}}} {w_i} }}$$

(4) Ensemble: Using ICHIO coding, the prediction results of each base learner are weighted and combined to form an integrated model, and the final prediction result *Y*_*pre*_ output by the model can be expressed as:

15 $$Y_{{pre}} = \mathop {\sum}\limits_{i = 1}^5 {W{}_i \times P_i}$$

here, *P*_*i*_ represents the prediction result output by the *i*th base learner. The validation set (10%) is predicted according to the integrated model, a 10-fold cross-validation is performed, and the 10-fold validation set prediction results and the real results are used to calculate the comprehensive error value.

(5) Objective function: The objective function is composed of error, L2 regularization of weight, and L1 regularization of feature quantity. The objective function can be expressed as

16 $$\min {{{\mathrm{ }}}}\frac{1}{m}\mathop {\sum}\limits_{i = 1}^m {(Y - Y_{{\rm{pre}}})^2} {{{\mathrm{ + }}}}\lambda \mathop {\sum}\limits_{i = 1}^{{{\mathrm{4}}}} {W_i^2} {{{\mathrm{ + }}}}\gamma \mathop {\sum}\limits_{i = 1}^{{{\mathrm{4}}}} {N_i}$$

here, *m* represents the number of samples, vector *Y* represents the actual output value, *Y*_*pre*_ represents the model predicted output value, and *λ* and *γ* are hyperparameters, which are set according to the data dimension (here, *λ* = 10, *γ* = 1).

### Evaluation metrics

The evaluation indexes for the classification model were obtained based on a confusion matrix, including the following four basic indexes: true positive (TP), indicating that the prediction is true and the outcome is true; true negative (TN), indicating that the prediction is false and the outcome is false; false positive (FP), indicating that the prediction is true and the outcome is false; and false negative (FN), indicating that the prediction is false and the outcome is true. On this basis, we used sensitivity (SEN), precision (PRE), specificity (SPE), accuracy (ACC), F1 score (F1), and area under the curve (AUC) to quantitatively evaluate the classification results. We chose the receiver operating characteristic-area under the curve (ROC-AUC) and precision recall-area under the curve (PR-AUC) as the preferred and comprehensive indicators. The value range of each index is [0–1]. The larger the value is, the better the classification effect.

The accuracy of a regression model can be evaluated by comparing the observed parameters with the estimated parameters. To compare the performance of the model, we used four common evaluation indexes: the mean square error (MSE), mean absolute error (MAE), root mean square error (RMSE) and goodness of fit (R-square, *R*^2^). The MSE and RMSE are used to measure the discreteness of a group of numbers, which are often controlled by larger values. The RMSE explains the error between the actual data and the predicted data. The MAE represents the actual prediction error. The first three indicators represent the model fitting error, while *R*^2^ represents the model fitting trend. The smaller the error value is, the better the fitting performance of the model. The value range of *R*^2^ is [0–1], which represents whether the prediction of the model follows the same trend as the actual data. The larger the value is, the stronger the ability of the model to predict the trend of the actual data^41^.

### Reporting summary

Further information on research design is available in the Nature Research Reporting Summary linked to this article.

## Supplementary information

Reporting Summary

## Data Availability

All data used in this study are publicly available through the sources referenced in the “Methods” section. The aggregated datasets analyzed in this study are available from the corresponding author on reasonable request.

## Code availability

Our code and data are publicly available at https://github.com/gccaizr/ICHIO.
